# The Effects of *Salvia miltiorrhiza* on Reproduction and Metabolism in Women with Polycystic Ovary Syndrome: A Systematic Review and Meta-Analysis

**DOI:** 10.1155/2021/9971403

**Published:** 2021-05-15

**Authors:** Wenjuan Shen, Bao Jin, Yaguang Han, Hongwei Wang, Huan Jiang, Linlin Zhu, Mei Han, Jiao Zhang, Yang Zhang

**Affiliations:** ^1^Department of Obstetrics and Gynecology, First Affiliated Hospital, Heilongjiang University of Chinese Medicine, Harbin 150040, China; ^2^Department of Obstetrics and Gynecology, Heilongjiang University of Chinese Medicine, Harbin 150040, China; ^3^Centre for Evidence-Based Chinese Medicine, Beijing University of Traditional Chinese Medicine, Beijing 100029, China; ^4^Department of National Physician Hall, Second Affiliated Hospital, Heilongjiang University of Chinese Medicine, Harbin 150009, China; ^5^Department of Internal Medicine, First Affiliated Hospital, Heilongjiang University of Chinese Medicine, Harbin 150040, China

## Abstract

**Objective:**

Polycystic ovary syndrome (PCOS) is the most common endocrine disorder in women of reproductive age. As a traditional medicine, *Salvia miltiorrhiza* (*S. miltiorrhiza)* has been widely used in the treatment of many gynecological diseases, but the efficacy of *S. miltiorrhiza* in women with PCOS has not been assessed. The purpose of this systematic review and meta-analysis was to evaluate the effectiveness and safety of *S. miltiorrhiza* in women with PCOS.

**Methods:**

We conducted searches in PubMed, Embase, the Cochrane Library, the China National Knowledge Infrastructure, the Wanfang Database, the Chinese Scientific Journal Database, and the Chinese BioMedical database from inception to December 23, 2020, to identify studies that met the inclusion criteria. The quality of the evidence was estimated using the Cochrane Reviewer Handbook 5.0.0, and the meta-analysis was performed using RevMan 5.3.5 software.

**Results:**

Six randomized controlled trials (RCTs) involving 390 patients with PCOS were included. The studies suggested that *S. miltiorrhiza* extract combined with letrozole (LET) was more effective in improving pregnancy rate (RR: 2.60, 95% CI: 1.06 to 6.39, *P*=0.04) compared to LET alone. *S. miltiorrhiza* extract was associated with decreased fasting blood glucose (MD: –0.25, 95% CI: –0.37 to –0.13, *P* < 0.0001), fasting insulin (MD: –1.16, 95% CI: –1.74 to –0.58, *P* < 0.0001), total cholesterol (TC) (MD: –0.58, 95% CI: –0.72 to –0.43, *P* < 0.00001), and triglycerides (TG) (MD: –0.31, 95% CI: –0.35 to –0.26, *P* < 0.00001) compared with placebo, but not with improvements in body mass index or waist-to-hip ratio (MD: –1.41, 95% CI: –4.81 to 2.00, *P*=0.42; MD: –0.02, 95% CI: –0.05 to 0.01, *P*=0.16, respectively). There was a significant difference between *S. miltiorrhiza* extract combined with cyproterone acetate (CPA) and CPA alone in terms of decreasing TC (MD: –0.77, 95% CI: –0.89 to –0.65, *P* < 0.00001), TG (MD: –0.43, 95% CI: –0.65 to –0.20, *P* < 0.0001), and low-density lipoprotein cholesterol (MD: –0.49, 95% CI: –0.66 to –0.33, *P* < 0.00001) and increasing high-density lipoprotein cholesterol (MD: 0.30, 95% CI: 0.20, 0.40, *P* < 0.00001). In addition, *S. miltiorrhiza* extract also decreased testosterone, follicle-stimulating hormone, and luteinizing hormone. The studies did not mention any adverse events with *S. miltiorrhiza* extract.

**Conclusion:**

The current studies indicate that *S. miltiorrhiza* has beneficial effects on reproduction and glucose and lipid metabolism in patients with PCOS, and it is generally safe for clinical application. However, more prospective RCTs with large samples, multiple centers, and longer intervention duration are needed in the future to obtain more reliable conclusions.

## 1. Introduction

Polycystic ovary syndrome (PCOS) is a heterogeneous endocrine disorder with a prevalence ranging from 6% to 21% in adolescent and reproductive age women depending on the diagnostic criteria that are used [1, 2]. Hyperandrogenemia and metabolic abnormalities are closely related to the occurrence and development of PCOS [3]. Excessive androgens can cause premature follicular atresia, and this results in ovulatory dysfunction and stimulates vigorous sebaceous gland metabolism, subsequently leading to infertility, menstrual disturbances, acne, hirsutism, and other clinical symptoms. More than 60% of PCOS patients will be accompanied by obesity, which can lead to dyslipidemia, abnormal secretion of adipokines, and abnormal steroid metabolism, and obesity further aggravates the occurrence and development of PCOS and increases the risk for type 2 diabetes, hyperinsulinemia, hyperlipidemia, and cardiovascular diseases [4, 5]. Studies have shown that PCOS patients are mainly concerned with reproductive disorders in the early stages of the disease but then become more concerned with the metabolic abnormalities in the later stages [6]. PCOS seriously affects the quality of life of patients and imposes a heavy burden on their families and society as a whole. A Bayesian modelling study in the UK in 2018 showed that women with PCOS require at least £237 million in treatment costs every year [7]. Thus, it is important to develop methods to improve the fertility of PCOS patients, reduce androgen levels, and correct metabolic disorders, and find ways to delay the development of the disease.

At present, western medicine (WM) treatments for PCOS mainly include ovulation-inducing drugs, especially combination oral contraceptives and insulin-sensitization agents, which act on different mechanisms to improve the pathological manifestations of PCOS, but these drugs cause abnormal uterine bleeding, weight gain, gastrointestinal discomfort, liver damage, and other side effects [8]. Thus, many PCOS patients seek complementary and alternative medicine treatments to enhance the efficacy of their treatments and to improve their health. Recent studies have shown that nutritional supplements and herbal medicines as complementary and alternative medicines may help improve the health status of women with PCOS [9], and there is a growing body of evidence that puerarin, semen cuscutae flavonoids, berberine, glycyrrhetinic acid, tanshinone, and other Chinese herbal extracts have advantages in the treatment of this disease [10]. *Salvia miltiorrhiza (S. miltiorrhiza)*, also known as danshen in traditional Chinese medicine, is used for the treatment of cardiovascular and cerebrovascular diseases [11, 12]. The active components of *S. miltiorrhiza* are divided into two groups: water-soluble phenolics [13] and lipophilic tanshinones [14]. Tanshinones (including tanshinone I, tanshinone IIA, tanshinone IIB, cryptotanshinone, and dihydrotanshinone I) exhibit diverse biological activities such as androgen reduction [15] and improvement in glucose and lipid metabolism [16, 17], and they have antitumor [18], cardioprotective [19], and neuroprotective [20] effects. Especially in the last decade, several clinical trials have evaluated the efficacy of *S. miltiorrhiza* for treating PCOS. Cryptotanshinone has been shown to reverse androgen excess and ovarian insulin resistance (IR) in mice by activating the insulin signaling pathway via PI3K and regulating glucose transporter and hormone synthase activity [21]. In addition, Yang et al. [22] demonstrated that cryptotanshinone can effectively reduce serum luteinizing hormone (LH) levels, the LH/follicle-stimulating hormone (FSH) ratio, and testosterone (T) levels, thereby reducing the expression of HMGB1, TLR4, and NF*κ*B/p65 in ovarian tissue and granulosa cells and improving the symptoms of PCOS.

Several randomized clinical trials (RCTs) have investigated the efficacy of *S. miltiorrhiza* in improving the pregnancy rate, fasting blood glucose, fasting insulin, body mass index (BMI), and T levels in patients with PCOS, but the results have been contradictory. Some reports indicated that the clinical pregnancy rate of PCOS patients is significantly increased after tanshinone capsule treatment [23]. However, Wang et al. [24] reported that the tanshinone capsule had no obvious effect on improving the pregnancy rate. In addition, other studies have shown that oral administration of tanshinone capsules in PCOS patients can reduce the fasting blood glucose level [13]. In contrast, Su et al. [25] did not show any effect of tanshinone capsule on fasting blood glucose levels in PCOS patients.

Due to the enthusiasm of PCOS patients for herbal therapy but the lack of strong evidence, evaluating herbal therapy is considered an appropriate research strategy because it may provide relevant answers to the questions of PCOS patients and clinicians. However, RCTs have shown contradictory reproductive and metabolic effects of *S. miltiorrhiza* in patients with PCOS. Moreover, the results of these RCTs may not be sufficient to draw firm conclusions in this regard. Therefore, in order to evaluate the effects of *S. miltiorrhiza* on the reproductive and metabolic outcomes of patients with PCOS more comprehensively and scientifically, we conducted a systematic review of the available RCTs in order to provide evidence-based medical guidelines for clinical practice.

## 2. Materials and Methods

This review was carried out according to the Cochrane recommendations and was reported according to the Preferred Reporting Items for Systematic Reviews and Meta-Analyses (PRISMA) statement [26].

### 2.1. Search Methods

Published articles were searched for in PubMed, Embase, the Cochrane Library, the China National Knowledge Infrastructure (CNKI), the Wanfang Database, the Chinese Scientific Journal Database (VIP), and the Chinese BioMedical database (CBM). The retrieval time was from the establishment of each database to December 23, 2020, and there were no restrictions on the languages nor publication status. Keywords in the literature retrieval included “*Salvia miltiorrhiza*,” “tanshinone,” “cryptotanshinone,” “tanshinone capsule,” “danshen,” “polycystic ovary syndrome,” “polycystic ovarian syndrome,” “PCOS,” “polycystic ovary changes,” and related synonyms.

### 2.2. Study Selection

All trials included in our study met the following criteria. (1) Participants were women diagnosed with polycystic ovary syndrome according to the Rotterdam criteria [27] or the recommendation of the American Androgen Excess Society [28]. There was no limitation on nationality, race, physical characteristics, or course of the disease. (2) We accepted RCTs regardless of blinding procedures but only included parallel design studies. (3) The interventions included *S. miltiorrhiza* extract or *S. miltiorrhiza* extract combined with WM (unlimited dosage form, dose, or duration). (4) The control group should be placebo, WM, or placebo combined with WM. (5) The study included the following outcome indicators: reproductive indicators (pregnancy rate); glucose and lipid metabolism indicators (fasting blood glucose, fasting insulin, total cholesterol (TC), triglycerides (TG), low-density lipoprotein cholesterol (LDL-C), and high-density lipoprotein cholesterol (HDL-C)); clinical symptoms (BMI, waist-to-hip ratio (WHR)); reproductive hormones (T, LH, and FSH); safety and adverse events data.

The exclusion criteria were as follows: (1) repeated publications or articles with unavailable data were excluded; (2) the article was an animal experiment, review, or case report; (3) other traditional Chinese medicine treatments such as acupuncture and massage were used; (4) trials were not RCTs or there were no criteria for how the trial was conducted.;(5) the full text could not be found.

### 2.3. Literature Screening and Data Extraction

The preliminary articles were imported into NoteExpressV3.3.0 for management. According to the inclusion and exclusion criteria, the titles and abstracts of the articles were read for preliminary screening after eliminating duplicated articles. The full text was then read during rescreening to identify the included articles. Data of each study were collected, including the following: study characteristics (primary author, publication year, study location, study design, and sample size), participant characteristics (mean age), intervention and comparison data (dose and treatment duration), and outcome measures. The results were extracted independently by two authors and then checked for consensus.

### 2.4. Quality Assessment

The methodological quality of the included RCTs was assessed for risk of bias in accordance with the RCT quality assessment criteria reported in the Cochrane Reviewer Handbook 5.0.0 [29] that focuses on the following six aspects: random sequence generation, assignment concealment implementation, blinding, data integrity, selective reporting with or without results, and other sources of bias, including whether there are clear inclusion/exclusion criteria, whether baseline data are comparable, and whether there is a conflict of interest. For each item, if satisfied, it means there is a “low risk of bias,” while contradicted items mean “high risk of bias.” When there is not enough information reported in the literature to allow one to make a clear judgment about an item, the item is classified as unclear, implying a moderate risk. The risk of bias for each qualifying study was independently assessed by two reviewers. If there was a disagreement, it was resolved through discussion or with the assistance of a third experienced researcher.

### 2.5. Data Analysis

RevMan 5.3.5 by the Cochrane Collaboration Network was used for meta-analysis. Binary variables are presented as the risk ratio (RR) with 95% confidence interval (CI), and continuous variables are expressed as the mean difference (MD) or standardized mean difference (SMD) with 95% CI. Heterogeneity was tested by the Q-test, in which *I*^2^ was used to quantitatively estimate the magnitude of heterogeneity. When *I*^2^ ≤ 50% and *P* ≥ 0.10, the fixed-effect model was used. If not, a random-effect model was used. If the heterogeneity was too large, the source of heterogeneity was identified and subgroup analysis or sensitivity analysis was conducted to determine the stability of the results of the meta-analysis.

## 3. Results

### 3.1. Literature Search Result

**Figure 1:**
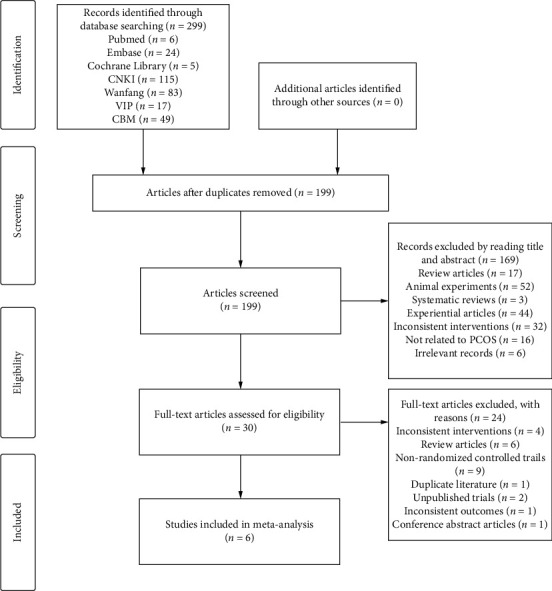
Flow diagram of the study selection process.

The initial search yielded 299 articles that met the search criteria, of which 115, 83, 17, 6, 49, 24, and 5 articles were from the CNKI, Wanfang, VIP, PubMed, CBM, Embase, and Cochrane Library, respectively. After removing duplicates, 199 articles remained. After scanning the titles and abstracts, we discarded 169 animal studies, evaluations, reviews, and clearly unqualified studies, leaving a total of 30 references for full-text review. After assessment according to the inclusion and exclusion criteria, 24 articles were further excluded, and 6 RCTs [23, 30–34] were included for analysis. The literature selection process is shown in Figure 1.

### 3.2. Features of the Included Literature

The six RCTs were published between 2015 and 2020 and had sample sizes ranging from 48 to 86, and the total number of involved patients was 390, including 195 in the intervention groups and 195 in the control groups. Two studies [32, 33] compared the efficacy of *S. miltiorrhiza* extract with placebo, and four studies [23, 30, 31, 34] compared the efficacy of *S. miltiorrhiza* extract in combination with WM versus WM alone. The characteristics of all the studies included in the meta-analysis are shown in Table 1.

### 3.3. Study Quality Assessment

**Figure 2:**
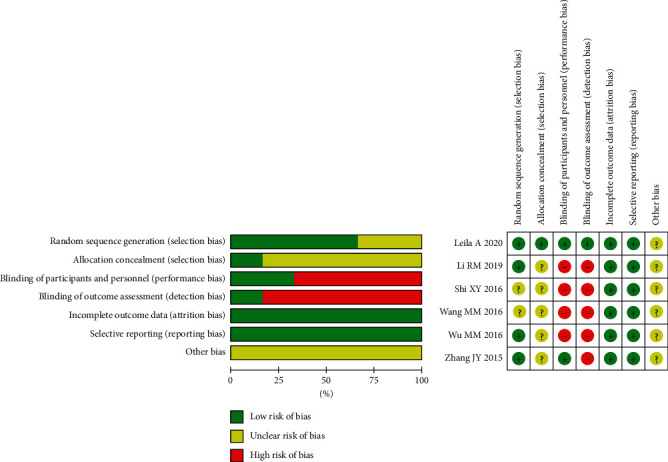
The risk of bias for the included studies.

Figure 2 summarizes the risk of bias of the six included RCTs. Four of the trials reported the random sequence generation methods, while the remaining two studies only mentioned “randomization” without describing specific randomization methods. Apart from Leila et al. 2020, the allocation concealment of the studies was not clearly defined. One study reported sample size estimates, and one study reported subjects falling off or being lost to follow-up. All studies were assessed as “low risk” for “selective reporting” because they reported the prespecified outcomes in the methods. Overall, the quality of six studies is low or remains unclear due to the high proportion of the unclear risk of biases in most studies.

### 3.4. Effects of *S. miltiorrhiza* on Pregnancy Rate

Two studies [23, 34] compared pregnancy rates and found no significant difference between the combination of *S. miltiorrhiza* extract + CPA versus CPA alone in improving the pregnancy rate [RR = 1.36, 95% CI (0.83, 2.21), *P*=0.22]. However, the combination of *S. miltiorrhiza* extract + letrozole (LET) was superior to LET alone in improving the pregnancy rate [RR = 2.60, 95% CI (1.06, 6.39), *P*=0.04].

### 3.5. Effects of *S. miltiorrhiza* on Reproductive Hormones

**Figure 3:**
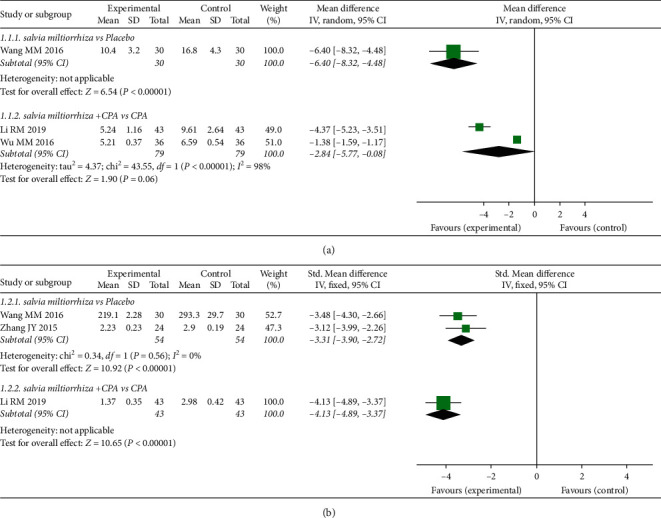
Meta-analyses of the effect of *S. miltiorrhiza* on reproductive hormones: (a) LH; (b) T.

*S. miltiorrhiza* extract showed a significant reduction of T [SMD = –3.31, 95% CI (–3.90, –2.72), *P* < 0.00001], LH [MD = –6.40, 95% CI (–8.32, –4.48), *P* < 0.00001], and FSH [MD = –5.70, 95% CI (–6.18, –5.22), *P* < 0.00001] versus the placebo group. Moreover, the combination of *S. miltiorrhiza* extract + CPA significantly decreased T [SMD = –4.13, 95% CI (–4.89, –3.37), *P* < 0.00001] and FSH [MD = –0.97, 95% CI (–1.59, –0.35, *P*=0.002], while LH was slightly decreased, but not significantly [MD = –2.84, 95% CI (–5.77, 0.08), *P*=0.06], in comparison to the CPA group (Figure 3).

### 3.6. Effects of *S. miltiorrhiza* on Glucose Metabolism

**Figure 4:**
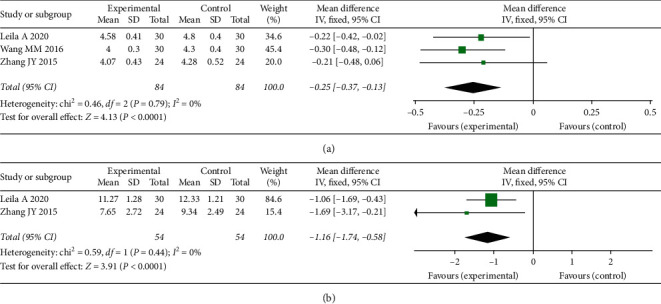
Meta-analyses of the effects of *S. miltiorrhiza* on glucose metabolism indexes: (a) fasting blood glucose; (b) fasting insulin.

Compared with the placebo group, PCOS patients treated with *S. miltiorrhiza* extract had significantly lower fasting blood glucose [MD = –0.25, 95% CI (–0.37, –0.13), *P* < 0.0001] and fasting insulin [MD = –1.16, 95% CI (–1.74, –0.58), *P* < 0.0001] (Figure 4).

### 3.7. Effects of *S. miltiorrhiza* on Lipid Metabolism

**Figure 5:**
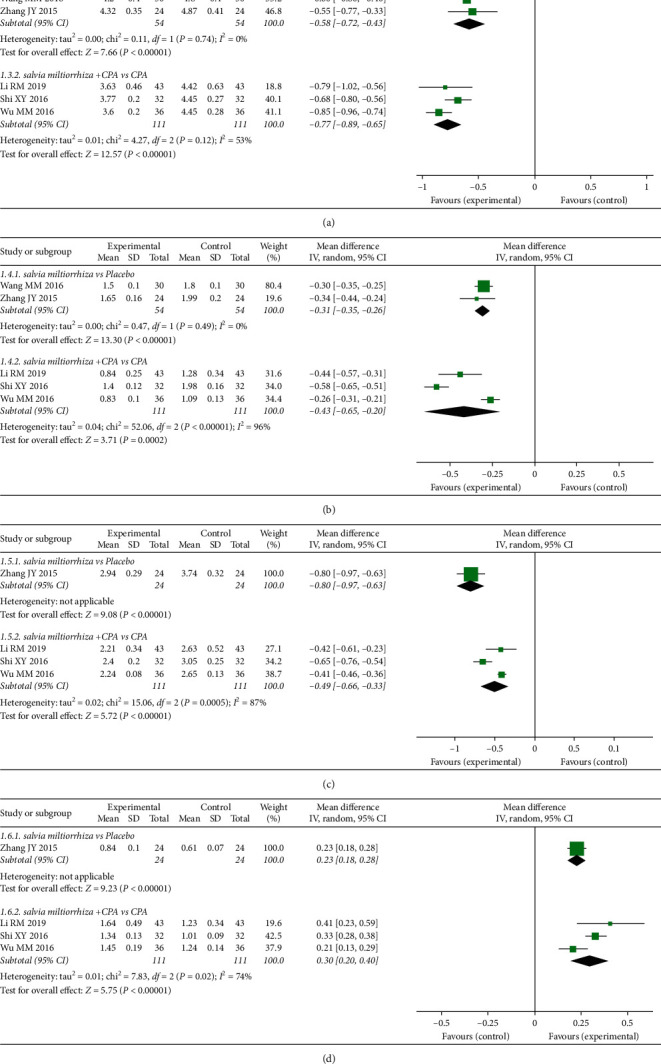
Meta-analyses of the effects of *S. miltiorrhiza* on lipid metabolism indexes: (a) TC; (b) TG; (c) LDL-C; and (d) HDL-C.

Two RCTs [23, 33] in the analysis compared the effect of *S. miltiorrhiza* extract and placebo on lipid metabolism in PCOS patients. As shown in Figure 5, the *S. miltiorrhiza* extract group was more effective in reducing TC [MD = –0.58, 95% CI (–0.72, –0.43), *P* < 0.00001], TG [MD = –0.31, 95% CI (–0.35, –0.26), *P* < 0.00001], and LDL-C [MD = –0.80, 95% CI (–0.97, –0.63), *P* < 0.00001] and increasing HDL-C [MD = 0.23, 95% CI (0.18, 0.28), *P* < 0.00001]. Our meta-analysis showed that the combination of *S. miltiorrhiza* extract + CPA significantly decreased TC [MD = –0.77, 95% CI (–0.89, –0.65), *P* < 0.00001], TG [MD = –0.43, 95% CI (–0.65, –0.20), *P* < 0.0001], and LDL-C [MD = –0.49, 95% CI (–0.66, –0.33), *P* < 0.00001] and increased HDL-C [MD = 0.30, 95% CI (0.20, 0.40), *P* < 0.00001] compared with CPA alone (Figure 5).

### 3.8. Effects of *S. miltiorrhiza* on Clinical Symptoms

**Figure 6:**
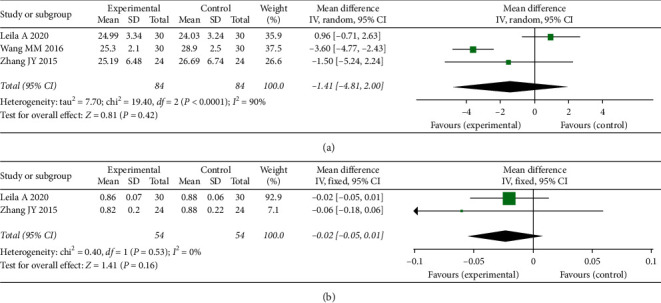
Meta-analyses of the effect of *S. miltiorrhiza* on (a) BMI and (b) WHR.

As shown in Figure 6(a), the three RCTs combined did not show any significant change in BMI in PCOS patients after treatment with *S. miltiorrhiza* extract versus placebo [MD = –1.41, 95% CI (–4.81, 2.00), *P*=0.42]. In addition, there was no evidence that *S. miltiorrhiza* extract was associated with improved WHR compared to placebo [MD = –0.02, 95% CI (–0.05, 0.01), *P*=0.16] (Figure 6(b)).

### 3.9. Safety Outcomes

Only the study by Zhang et al. reported no adverse events, and the other studies did not report any information about adverse events.

### 3.10. Sensitivity Analysis

Sensitivity analysis revealed that excluding individual studies did not remarkably influence the overall effect size of TC, TG, LDL‐C, and HDL‐C. In addition, removing Wu et al. [30], which reported on the effect of *Salvia miltiorrhiza* extract on lipid metabolism, resulted in a decrease in heterogeneity, whereas the result remained significant, TC (95% CI [–0.81, –0.60], *I*^2^ = 0%), TG (95% CI [–0.66, –0.38], *I*^2^ = 72%), LDL-C (95% CI [–0.77, –0.32], *I*^2^ = 77%), and HDL-C (95% CI [0.28, 0.39], *I*^2^ = 0%).

## 4. Discussion

To the best of our knowledge, this systematic review of six RCTs is the latest and most comprehensive data analysis of *S. miltiorrhiza* in the treatment of PCOS so far, and it was designed to evaluate the reproductive and metabolic effects of *S. miltiorrhiza* in women with PCOS. Compared with the control group, *S. miltiorrhiza* extract may improve pregnancy rate; decrease T, FSH, fasting blood glucose, fasting insulin, TC, TG, and LDL-C; increase HDL-C. However, there is no strong evidence that *S. miltiorrhiza* extract has an effect on BMI and WHR. For patients with PCOS, excessive androgen and insulin resistance can reduce endometrial function leading to decreased fertility [35–37]. Based on current evidence, *S. miltiorrhiza* may be recommended for the treatment of PCOS patients with a desire for fertility and/or those with hyperandrogenism and metabolic disorders.

Based on our results, oral *S. miltiorrhiza* extract appears to have an effect on the pregnancy rate in PCOS patients. *S. miltiorrhiza* may improve fertility through various possible mechanisms. For example, when the endometrial microcirculation is disturbed, the implantation rate is low, and *S. miltiorrhiza* can modify endometrial microcirculation-related indicators, such as the endometrial pulsatility index and resistance index, and thus improve endometrial receptivity to provide a good environment for embryo implantation [38, 39]. It also improves the efficacy of PMSG for increasing the pregnancy rate [40]. In addition, traditional Chinese medicine decoctions containing *S. miltiorrhiza* have been shown to improve reproductive outcomes by regulating the expression of proteins such as integrin, vascular endothelial growth factor, and uncoupling protein 2, which are closely related to endometrial receptivity [41]. Hyperandrogenemia is the main endocrine characteristic of PCOS. Hyperinsulinemia and IR are considered to be the main causes of hyperandrogenemia [35, 36], while excessive androgen leads to increased levels of LH and FSH [37]. In PCOS, theca cells and granulosa cells overexpress mRNA encoding enzymes involved in steroidogenesis, including androgen receptor, CYP11, CYP17, and CYP19, and this can lead to disturbances in ovarian hormone synthesis. The recent studies have shown that *S. miltiorrhiza* extract can reverse reproductive disorders by regulating the expression of androgen receptor, CYP11, CYP17, and CYP19, thus improving reproductive hormone production such as T, LH, and FSH [15, 21].

Another important result to take into consideration is the positive effect of *S. miltiorrhiza* extract on lipid profiles in PCOS patients. Animal and experimental studies have shown that *S. miltiorrhiza* has antiobesity effects, and in vitro studies have shown that *S. miltiorrhiza* inhibits adipogenesis in 3T3-L1 preadipocytes, with this inhibition mainly occurring at an early phase of adipogenesis through the attenuation of mitotic clonal expansion via cell cycle arrest at the G1/S phase transition [42]. It also can suppress adipogenesis and reduce obesity-related metabolic disorders by acting on PPAR*γ*, C/EBP*α*, GATA-2, and GATA-3 [43]. Tanshinone IIA also suppresses fatty acid-induced lipogenesis and TG accumulation in HepG2 cells [44], and a recent study has shown that *S. miltiorrhiza* significantly decreases TC, TG, and LDL-C levels in mice by inhibiting the expression of FAS mRNA and HMGR mRNA [45]. Furthermore, *S. miltiorrhiza* extract reduces the upregulation of SREBP1 and TG induced by high glucose in LO2 cells, which improves lipid metabolism, and the underlying mechanism is probably through the regulation of STAT-3 signaling [46]. Lastly, *S. miltiorrhiza* can activate the estrogen receptor through the ERK signaling pathway, reducing lipid deposition in the aorta [16].

Beyond its effects on lipid profiles, *S. miltiorrhiza* has positive effects on other aspects of PCOS patients' health. PCOS is closely related to metabolic syndrome [47, 48], and several studies have demonstrated the different effects of *S. miltiorrhiza* on hypoglycemia and hypoinsulinemia. Adiponectin (APN) and leptin are closely related to obesity [49]. The imbalance of adipose cytokines such as leptin and adiponectin secreted by adipose tissue in obese patients also aggravates the IR associated with PCOS [50]. *S. miltiorrhiza* extract increases the sensitivity to insulin by inducing the production and secretion of anti-inflammatory adipokines (such as APN), and it reduces inflammation and the production of proinflammatory cytokines [51, 52]. Tanshinone I may also decrease fasting blood glucose concentrations by decreasing levels of interleukin-6 and tumor necrosis factor-alpha and by reducing the nuclear translocation of NF-*κ*B and the phosphorylation of Ser307 on insulin receptor substrate 1 (IRS-1) [42]. It is also suggested that the hypoglycemic effect of *S. miltiorrhiza* extract is likely to be secondary to an action on carbohydrate metabolism [53]. The observed decrease in fasting blood glucose and fasting insulin concentrations may be a result of cryptotanshinone rescuing the altered protein expressions of IRS-1 and IRS-2, phosphatidylinositol 3-kinase p85*α*, glucose transporter-4, ERK-1, and 17*α*-hydroxylase [54]. Lastly, cryptotanshinone stimulates insulin signaling and the regulation of glucose transporters and hormone-synthesizing enzymes, which reverses ovarian IR in mice [21].

There is no strong evidence in our analysis that *S. miltiorrhiza* extract has effects on BMI and WHR. The only exception was the study by Wang et al. [23], who found that the *S. miltiorrhiza* extract group significantly reduced BMI compared with placebo.

Among the included studies, Zhang et al. [33] reported that no adverse reactions occurred during the treatment, such as gastrointestinal discomfort, skin rashes, dizziness and headaches, blood system changes, and liver and kidney damage, while the other studies did not mention anything about adverse events at all. In addition, a more recent study [55] has shown that *S. miltiorrhiza* has the advantages of strong activity, low toxicity, low side effects, and extensive pharmacological effects. Therefore, we believe that *S. miltiorrhiza* is a relatively safe treatment, although more clinical studies are needed to confirm the safety of *S. miltiorrhiza* in long-term treatment.

This review included six recent RCTs (2015–2020), and limitations include the small number of studies, the small sample sizes, the lack of blinding in most of the studies, missing descriptions of the randomization method in some studies, and results with high heterogeneity (e.g., TC, TG, LDL-C, and HDL-C). Bias risk sensitivity analysis was used to investigate the high heterogeneity. After the study by Wu et al. was excluded, TC and HDL-C showed homogeneity. The heterogeneity might therefore be related to the place where the experiment took place, the subjects' dietary habits, and their own constitutions. The interventions included in this study included *S. miltiorrhiza* extract combined with CPA or LET. CPA was used as a combination oral contraceptive and LET was used as an ovulation-induction drug. The mechanism of action and the goal in clinical application of the two are different, so the data from these studies were not combined. The study time was generally short to medium term (mostly 3 months), and there was a lack of follow-up observation of the long-term efficacy of *S. miltiorrhiza*. In addition, the adverse effects of *S. miltiorrhiza* on PCOS are unknown because most trials did not report adverse events, and caution should be used in interpreting the safety of *S. miltiorrhiza*. Finally, we still need a large sample, multiple center, and scientifically validated RCTs to further verify the efficacy of *S. miltiorrhiza* in the treatment of PCOS and to provide solid and reliable evidence for clinical practice.

## 5. Conclusion

In summary, the results of the present systematic review and meta-analysis indicate that *S. miltiorrhiza* has beneficial effects on reproduction and glucose and lipid metabolism in patients with PCOS. Moreover, our results demonstrate that the clinical application of *S. miltiorrhiza* is generally safe. However, due to the relatively low quality of the included studies, we urge caution in promoting these results. More prospective RCTs with large samples, multiple centers, and longer intervention durations are warranted in the future to obtain more scientific, objective, and reliable conclusions.

## Figures and Tables

**Table 1:** The details of the included studies.

Authors/published year	Study location	Study design	Sample size (T/C)	Mean age (T/C)	Intervention	Comparison	Dosage	Duration of treatment	Outcomes
Zhang et al. 2015 [33]	Jiangsu Province, China	RCT	24/24	21–34/20–31	*S. miltiorrhiza* extract	Placebo	100 mg, tid	3 months	BMI, WHR, fasting blood glucose, fasting insulin, TC, TG, HDL-C, LDL-C, T
Amini et al. 2020 [32]	Tehran, Iran	RCT	30/30	28.07 ± 4.18/29.23 ± 5.44	*S. miltiorrhiza* extract	Placebo	330 mg, qd	8 weeks	BMI, WHR, fasting blood glucose, fasting insulin
Wu et al. 2016 [30]	Xinjiang Uygur Autonomous Region, China	RCT	36/36	28.2 ± 4.5	*S. miltiorrhiza* extract + CPA	CPA	*S. miltiorrhiza* extract 100 mg, tid CPA 1 pill, qd	3 months	TC, TG, HDL-C, LDL-C, LH
Li 2019 [31]	Henan Province, China	RCT	43/43	29.87 ± 4.23/30.02 ± 4.51	*S. miltiorrhiza* extract + CPA	CPA	*S. miltiorrhiza* extract 100 mg, tid CPA 1 pill, qd	3 months	TC, TG, HDL-C, LDL-C, T, LH, FSH
Shi et al. 2016 [34]	Sichuan Province, China	RCT	32/32	25.1 ± 3.8/25.0 ± 4.1	*S. miltiorrhiza* extract + CPA	CPA	*S. miltiorrhiza* extract 100 mg, tid CPA 1 pill, qd	2 months	Pregnancy rate, TC, TG, HDL-C, LDL-C
Wang et al. 2016 [23]	Jiangsu Province, China	RCT	30/30	18–37/20–38	*S. miltiorrhiza* extract + LET	Placebo + LET	*S. miltiorrhiza* extract 100 mg, tid LET NR	3 months	Pregnancy rate, BMI, fasting blood glucose, TC, TG, T, LH, FSH

CPA: cyproterone acetate; LET: letrozole; BMI: body mass index; WHR: waist-to-hip ratio; TC: total cholesterol; T: testosterone; TG: triglycerides; FSH: follicle-stimulating hormone; LH: luteotropic hormone; HDL-C: high-density lipoprotein cholesterol; LDL-C: low-density lipoprotein cholesterol; NR: not reported.

## Data Availability

The data used to support the findings of this study are available from the corresponding author upon request.
